# Public and professional involvement in a systematic review investigating the impact of occupational therapy on the self-management of rheumatoid arthritis

**DOI:** 10.1177/03080226231219106

**Published:** 2023-12-30

**Authors:** James P Gavin, Laura Rossiter, Vicky Fenerty, Jenny Leese, Jo Adams, Alison Hammond, Eileen Davidson, Catherine L Backman

**Affiliations:** 1 School of Health Sciences, University of Southampton, Southampton, UK; 2 Library Services, University of Southampton, Southampton, UK; 3 Arthritis Research Canada, Vancouver, BC, Canada; 4 School of Epidemiology and Public Health, University of Ottawa, Ottawa, ON, Canada; 5 School of Health and Society, University of Salford, Salford, UK; 6 Department of Occupational Science and Occupational Therapy, University of British Columbia, Vancouver, BC, Canada

**Keywords:** Patient and public involvement, GRIPP2, rheumatoid arthritis, systematic review, rheumatology

## Abstract

**Introduction:**

Public and health professional involvement (PHPI) is essential in healthcare research yet uncommonly integrated into systematic reviews. We incorporated and evaluated PHPI in a mixed methods review of occupational therapy for self-management of rheumatoid arthritis (RA).

**Methods:**

Public partners were living with or caring for someone with RA. Our steering group comprised two public, two professionals (one occupational therapist, one rheumatologist), and one reviewer who planned the review’s PHPI (August 2021). Involvement was evaluated from public and health professional (PHP) perspectives using a survey and workshops (August–October 2022) exploring reasons for involvement, challenges and learning opportunities.

**Results:**

Alongside the steering group, 16 public and 6 professionals were involved throughout the review. Five public refined the search strategy, with three assisting in subsequent review activities. PHPs helped interpret findings during three public (*n* = 12) and one professional workshop (*n* = 4). Three occupational therapists and one public co-authored (ED) publications. In evaluation, PHPs felt valued and that their involvement was well-integrated. The researchers underestimated the time required for communicating and conducting PHPI in the review.

**Conclusions:**

PHPI is worthwhile, feasible and can be integrated within a systematic review. PHP partners considered participation valuable; researchers must prioritise time to prepare and communicate PHPI activities.

## Introduction

Public and patient involvement (PPI) is integral to healthcare research and mandated by funding and commissioning bodies in many developed nations (Bastian, 1998; Entwistle and O’Donnell, 2003; Tembo et al., 2019; de Iongh et al., 2021; Tricco et al., 2022). The United Kingdom (UK) National Institute of Health Research (NIHR) defines PPI in research as an active and informative partnership, whereby research is conducted ‘*with*’ or ‘*by*’ the public (i.e. patients, service users and/or carers), rather than ‘*about*’ or ‘*done*’ to them (NIHR INVOLVE, 2012). PPI enhances research quality and relevance by aligning it with patients’ needs and issues of importance. However, there are challenges to public involvement. For example, ‘tokenism’ in making symbolic or superficial efforts for involvement, such as recruiting public partners to satisfy project/funder requirements without renumeration for their time, invitation or proper consideration to collaborate on short- or long-term tasks (Leese et al., 2018). The UK Standards for PPI promote good practice for investigators implementing this in research (Tembo et al., 2019; The UK Public Involvement Standards Development Partnership, 2020). Key principles are inclusion, collaboration and co-production as equal partners, which align with the professional tenets of occupational therapy (Harries et al., 2020). A priority of occupational therapy research is to support meaningful occupations within and throughout an individual’s life, their communities and wider society (Nayar and Stanley, 2015). Involving service users and the public in research is fundamental to client-centred occupational therapy practice (Hammell and Iwama, 2012; Røssvoll et al., 2022). Understanding and evaluating what works and does not work in PPI for occupational therapy research contributes to the development of the profession’s evidence base.

There is scant detail about what constitutes ‘*good*’ in effective and successful PPI in different research methods (Liabo et al., 2020; McCoy et al., 2018). This is partly attributable to the shift, in the last 20 years, from ‘*why*’ the public should be involved to ‘*how*’ to best involve them. Reporting of PPI in research may be limited by article length requirements, leading to underestimates of PPI in previous healthcare research (Price et al., 2018; Williamson et al., 2023). The Guidance for Reporting Involvement of Patients and the Public 2 (GRIPP2) framework (Staniszewska et al., 2017) was developed to improve the quality and consistency of reporting PPI in research articles and subsequently increase the research community’s understanding of how research works, in what context/setting, for whom and why (Jones et al., 2021; Williamson et al., 2023). However, given that PPI increasingly features in health research (Prior et al., 2022), the complexity is in the ‘*how*’, which components are effective, what can be improved and whether health inequalities are recognised (de Iongh et al., 2021). This has been found to be scarce in occupational therapy (Williamson et al., 2023).

Although research evaluating the methods and benefits of PPI in research exists (Domecq et al., 2014; Greenhalgh et al., 2019; Wilson et al., 2015), there are fewer examples of reporting and/or evaluating this in systematic reviews in occupational therapy (Backman et al., 2022; Williamson et al., 2023). One such example is a recent scoping review by Røssvoll et al. (2022) exploring how PPI has been conducted and evaluated in occupational therapy research. PPI varied across 17 studies (14 qualitative), with evaluations predominantly positive for co-production in research methods and ethics by public partners, and researchers. However, all evaluations were anecdotal and only five reported benefits to the public partners and researchers. Few systematic reviews have involved PPI throughout the entire research cycle (Jones et al., 2021; Vale et al., 2012), with most only integrating PPI via project steering committees (Kiely et al., 2022; Pollock et al., 2015). For example, Brütt et al. (2017) engaged patients in planning a systematic review of metacognitive interventions for mental health and subsequently involved them in ranking the review outcomes. Although limited, involvement was deemed a positive experience by the patients. The researchers recommended involving PPI throughout the review cycle, from co-producing the review question and methods to interpreting the review findings and dissemination. This is particularly important for systematic reviews as they are critical tools for rigorously evaluating intervention impacts and informing the design of future healthcare interventions.

Between 2020 and 2021, the Royal College of Occupational Therapists (RCOT) ran a Priority Setting Partnership with the James Lind Alliance to determine the issues that matter most to people using occupational therapy services and professionals delivering occupational therapy. The primary priority, ‘*how does occupational therapy make a difference and have an impact on everyday lives?*’ showed clear value in interventions being evaluated in terms of a client’s lived experience, and clinical and cost-effectiveness. Qualitative evidence (e.g. self-reported accounts of lived experience) can complement quantitative evidence (e.g. ‘objective’ numerical estimates of pain and function) in capturing an individual’s experience of undergoing a particular treatment/intervention (Mcfeely, 2022; Prior et al., 2022). Potential benefits of such mixed methods research include greater insights into complex interventions, better decision-making and greater impact on daily lives (i.e. client-centredness) (Creswell and Clark, 2017).

The involvement of healthcare professionals in research has potential to enhance care provision, even though not the primary research aim (Boaz et al., 2015). Various mechanisms can explain improved health provision, including changing professional attitudes/behaviours, developing clinical-academic networks and offering opportunities for contemporising knowledge. Recently the Council for Allied Health Professions Research published: Shaping Better Practice Through Research: A Practitioner Framework (Harris et al., 2020) to support allied health professionals conducting and collaborating in research. A recent systematic review identified beneficial impacts of involving health professionals encompassing: patients (in improved care); service provision and workforce (improved service delivery/career pathways); culture and capacity; attracting research funding; knowledge exchange to upskilling healthcare professionals (Newington et al., 2021).

In August 2021, we began a mixed methods systematic review on the impact of occupational therapy in the self-management of rheumatoid arthritis (RA), including quantitative (e.g. randomised controlled trials) and qualitative findings (e.g. case reports and mixed methods studies) across peer-reviewed and grey literature. Previous reviews on the topic were limited to Level 1, quantitative evidence between 2002 and 2014 (Siegel et al., 2017; Steultjens et al., 2004), without consideration of people’s lived experiences of self-management. Implicit in the traditional hierarchy of research methods for evidence-based practice is that quantitative methods are superior to qualitative methods (Burns et al., 2011). Although this assumption is challenged in contemporary research, it partly explains the lack of consideration for and inclusion of qualitative evidence in previous reviews. Our recent interview study produced valuable insights into people’s experiences of self-care for their RA during the COVID-19 pandemic, relating to personal adaptation, emotional management and changing communication with health professionals (Leese et al., 2022). Our team, including our public and health professional (PHP) partners, therefore considered it crucial to include qualitative and quantitative evidence in our review.

Public and health professional involvement (PHPI) in our review was informed by Harries et al. (2020) considerations for occupational therapists in undertaking people-centred research. In particular, ‘*how are the experiences of public participants captured?*’; ‘*how can we seek feedback from a broad range of individuals (i.e. public and professionals)*?’; ‘*how have the public impacted our research design?’; ‘how do we show those involved that their contributions are valued?’* and *‘where is the reciprocity within our relationships with service users who inform our research?’* using the GRIPP2 framework (Staniszewska et al., 2017). Adhering to these, we collaboratively involved people with RA, carers (partners/family), and health professionals in a mixed methods systematic review to assess the role of occupational therapy in the self-management of RA for quantitative (e.g. pain, function, fatigue) and qualitative (self-reported accounts of lived experience) outcomes (Gavin et al., 2022). We aimed to incorporate and evaluate PHPI throughout the systematic review study.

**Figure 1.**
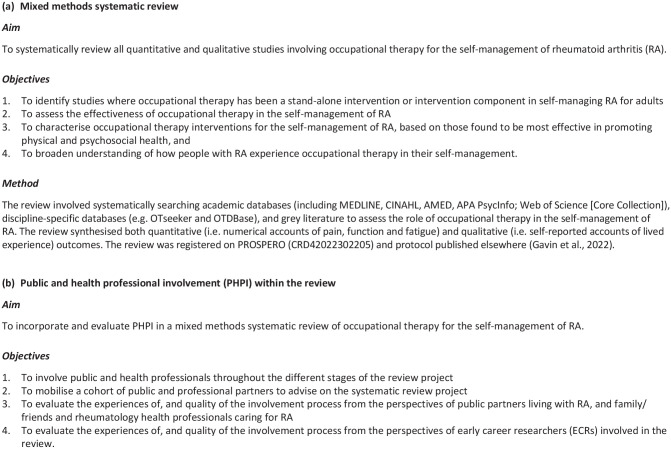
(a) Aim, objectives and summarised methods for the mixed methods systematic. (b) Aim and objectives for public and health professional involvement within the review.

As background to this PHPI work, Figure 1 summarises the systematic review’s aim and objectives. On completion, the experiences of, and quality of the involvement process, were evaluated from the perspectives of service users, professionals and early career researchers (ECRs) involved in the review project. This allowed us to inform occupational therapy and systematic review research in general, and our future research.

## Method

### Research design

In August 2021, our team of interdisciplinary researchers set out to involve people living with RA and rheumatology healthcare professionals in a mixed methods systematic review (Gavin et al., 2022). The PHPI was facilitated in the review via (i) a project steering group, (ii) a co-investigator with lived experience (ED) and (iii) the PHP partners. Experiences of the three ECRs within the research team were also sought. Individuals were considered ‘public’ stakeholders if they were living with RA, or a partner/friend caring for someone with RA. Involved healthcare professionals were registered occupational therapists or rheumatologists caring for those with RA. The co-investigator with lived experience (ED) is a RA advocate (https://chroniceileen.com/) and member of the Arthritis Research Canada Patient Advisory Board, who collaborated with the research team before the review project and co-authored the funding bid (January–February 2021). ED was a partner at all stages of the review, co-authoring publications and in follow-on research. Figure 1 summarises the method for the mixed methods systematic review.

To evaluate the quality of the PHPI process from public, professional and academic perspectives, we conducted an online survey. We purposively sampled PHPs in the UK, and the three ECRs of the research team, who were involved in all phases of the systematic review project (co-authors JG, LR and JL). Two to three months later, online evaluation workshops were held with those volunteering from the public and professional samples only. The survey and workshops occurred between the review interpretation and dissemination phases (June 2022–February 2023). Public partners, rheumatology occupational therapists, and the three ECRs of the research team completed evaluations.

### The project steering group

Firstly, the steering group was created comprising: two women living with RA (aged 50 and 61 years, one with a mixed British/Latin background (RA duration 6 years) and one with a White Scottish background (RA duration 35 years), who was also the Chair of the Versus Arthritis’ Patient Insight Partner group); an occupational therapist; a consultant rheumatologist and one former systematic reviewer for the Southampton Health Technology Assessment Centre. Their role was to advise on the review process and PPI planning throughout the 12-month project. Three steering group meetings were held during the project to inform: (i) planning the review (September 2021), (ii) the search strategy (January 2022), and (iii) the synthesis and interpretation of results (May–July 2022). For dissemination, correspondence was via email and online calls with individual group members.

### Establishing a cohort of public and professional partners

To support the steering group, we created a network of public (*n* = 13) and professional (*n* = 3; occupational therapists) partners who had lived experience of, and accessed health care services for their clinically diagnosed RA, or cared for others with RA. Recruitment was via NHS People in Research (https://www.peopleinresearch.org/) (~35–45%) and professional networks of the research team and project steering group (~55–65%). Individuals interested in becoming partners contacted the principal investigator (JG), who explained the review aims, timeline and PPI activities. Public partners (*n* = 13; 11 women, two men (aged 38–74 years)) from across the UK were invited to partake in online workshops to (i) refine the search strategy (September 2021), (ii) interpret the review findings from a lay perspective (June–August 2022) and/or (iii) evaluate the project (February 2023). Health professionals were occupational therapists and rheumatologists recruited via the professional networks of the research team, the RCOT and the British Society of Rheumatology. Individuals were emailed the review summary and involvement activities by the principal investigator (JG), and follow-up meetings were scheduled online using Microsoft Teams. One additional rheumatology occupational therapist became a professional partner for the interpretation phase only, complementing the three professional partners who were leads of the RCOT specialist section, Trauma and Musculoskeletal Health/Rheumatology Clinical forum.

### Involvement of public partners: Refining the search strategy

Online workshops were held to refine the search strategy and then interpret the review findings. These workshops were facilitated by two reviewers (JG and LR), guided by a semi-structured interview schedule and lasted ~90 minutes. The search strategy workshop involved a randomly-invited selection of public partners (*n* = 5) being presented with the initial search terms for the review, based on the SPIDER framework (see Figure 1; Methley et al., 2014). Each partner reviewed our initial search terms relating to: physical, psychological, social, emotional and intellectual ‘outcomes’ (including self-care concepts), ensuring that these resonated with their lived experiences. The healthcare professionals were not involved in this stage, but the initial search terms were formalised by the multidisciplinary research team (i.e. the principal investigator (JG, a physiologist), clinical academics specialising in occupational therapy and rheumatology (CB, JL, JA and AH), a health sciences librarian (VF) and graduate occupational therapist (LR)). Thereafter, the review was conducted by JG and LR, with JL providing subject-specific expertise for qualitative and mixed methods articles.

### Involvement of public and professional partners: Interpreting the review findings

To increase understanding of the review results from PHP perspectives, four, 90-minute online workshops were convened between June and August 2022. Three workshops involved public partners (i.e. living with, or caring for RA) (*n* = 12), one workshop involved rheumatology occupational therapists (*n* = 4). Workshops were facilitated by two ECR reviewers (JG and LR), who presented a summary of the review results to PHP partners. The full, detailed results were emailed to attendees at least 72 hours before the workshop. Articles included in the review were classified under four intervention types: (i) patient education; (ii) behaviour change; (iii) comprehensive, community (home) occupational therapy – quantitative and qualitative and (iv) other interventions (including exercise and workplace interventions), with a particular focus on what is implemented in real-life occupational therapy and RA self-management. Each workshop ended with public or professional partners discussing how the review findings could inform future research and practice. Workshops were not recorded, as their aim was PHP involvement, not participation in research. Field notes were taken to ensure individual narratives were captured in real-time and subsequently typed up with individuals pseudonymised. All public partners were reimbursed for their time per recommended NIHR rates (NIHR, 2022).

### Evaluation of public, health professional and ECR involvement

One month after each interpretation workshop, PHP were emailed an evaluation form to complete (via Google Forms; July–September 2022). Finally, as the survey was completed anonymously, all were again invited to discuss their experiences further, during an online evaluation workshop led by the principal investigator (JG; October–November 2022).

**Figure 2.**
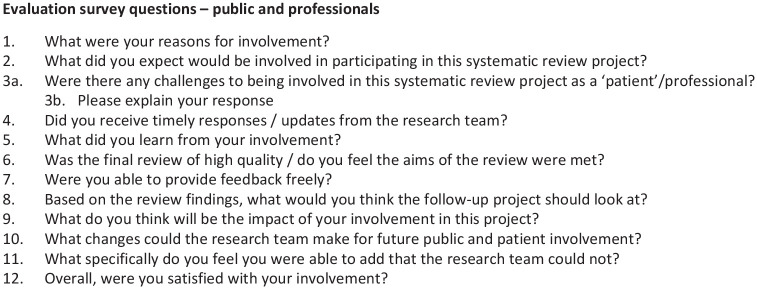
Public and professional involvement in a mixed methods systematic review – evaluation survey. Note: Eight respondents (n=6 public, n=2 professional). Evaluation survey submitted anonymously.

Survey questions were adapted from Vale et al. (2012), focusing on: reasons for involvement; challenges to involvement; learning opportunities; communication with the research team (including feedback provision); the impact of involvement and added value from involvement, with an additional focus on the impact of this occurring during the COVID-19 pandemic (Figure 2). The survey questions acted as discussion points during each workshop (held 3–4 weeks after), allowing participants to expand upon their survey responses. An independent researcher, not involved in the systematic review, helped create an ECR evaluation survey. This was informed by PHP survey questions, but for the ECR reviewers (JG, LR and JL) to complete based on their understanding of PHPI (on beginning the systematic review), barriers to involvement (during and post-review) and lessons learnt (post-review) (Supplemental Figure 1). The ECR co-authors were involved throughout the systematic review, from identifying the review question (JG and JL), to developing and piloting the search strategy, to conducting the review, to the interpretation and dissemination phases (JG, LR and JL). The ECR survey was, therefore, completed later in December 2022. Basic content analysis was conducted by the principal investigator (JG) for both PHP and ECR responses, used to collate responses to questions and identify major themes (herein ‘topics’, given this article reports PHPI in research, and not the research findings) and trends arising from the responses (Terry et al., 2017). Topics were *feeling valued and heard; sharing and learning; communication and listening for partnership building*. Lastly, a topic summary was devised and emailed to PHP partners, along with the plain English summary of the systematic review. They were invited to comment on the preliminary topics and offer additional feedback on their involvement with the study.

## Results

### Summary of public and professional involvement

#### Refining the search strategy

The search strategy was refined by five people with RA during a 90-minute online workshop; four of these subsequently contributed to the interpretation and evaluation phases (Table 1; Supplemental Figure 2). The initial search strategy, devised by the research team for the review protocol, was complemented with the following terms: *body image (or self-esteem or self-image); health literacy; wearable electronic devices; mobile applications; tai chi; meditation; hydrotherapy; postural balance; dependency; psychological; social stigma; social isolation* (Gavin et al., 2022).

**Table 1.** Characteristics of public partners, health professional partners and early career researchers in the review team.

Participant	Activity					
	Project steering group (*n* = 2 with RA)*	Cohort of PHP partners (*n* = 16)	Refining the search strategy (*n* = 5 with RA)	Interpreting the review findings (*n* = 16)	Evaluation (survey)^†^ (*n* = 11)	Evaluation (workshop) (*n* = 7)
**Public with/carers for RA** (gender, age, ethnicity [region], RA duration)						
Female, 50 years, mixed British/Latin (Surrey), 6 years	✓	✓	✓	✓	n/d	✓
Female, 50s, white Scottish (Greater Manchester), 35 years	✓	✓	✓	✓	n/d	✓
Female, 56 years, London (south-east), 11 years		✓		✓	n/d	
Male, 74 years, white British (East Midlands), 26 years		✓		✓	n/d	
Female, 62 years, white British (East Midlands), 20 years		✓	✓	✓	n/d	✓
Male, 29 years, British Indian (London), n/d		✓		✓	n/d	
Female, 52 years, British Indian (West Yorkshire), carer		✓		✓	n/d	
Female, ~50 years, white British (West Midlands), 9 years		✓	✓	✓	n/d	
Male, ~55 years, British Indian (Greater Manchester), carer		✓		✓	n/d	✓
Female, ~45 years, white British (Hampshire), ~10 years		✓			n/d	
Female, 44 years, British Indian (Greater London), 13 years		✓		✓	n/d	
Female, n/d, British Asian (Leicestershire), 20 years		✓	✓	✓	n/d	✓
Female, 52 years, white Scottish (Hampshire), ~8 years		✓		✓	n/d	
*Total*	2	13	5	12	6	5
**Healthcare professionals** (gender, ethnicity [region], NHS level, years in rheumatology)						
Female, white Scottish (Lanarkshire, Scotland), Band 7 – Advanced Practitioner, 4 years		✓		✓	n/d	✓
Female, white British (north-west England), Band 7 – Advanced Clinical Practitioner/Hand Therapist, 15 years		✓		✓	n/d	✓
Female, white British (Fife, Scotland), Head of Occupational Therapy Rheumatology, 29 years		✓		✓	n/d	
Female, white British (south-west), n/d, n/d				✓	n/d	
Female, white Irish (south-west), Advanced Practitioner / Hand Therapist, 5–10 years	x				n/d	
*Total*	0	3	0	4	2	2
**Early career researchers** (gender, ethnicity (region), specialism, age)						
Male, white British (south-west), Lecturer (musculoskeletal health), 36 years				✓		
Female, white British/Canadian/Irish (British Columbia, Canada), Post-doctorate (knowledge translation), 38 years				✓		
Female, white British (south–west), Graduate occupational therapist (mental health/work), 26 years				✓		
*Total*					3	

* Only two members of the steering group are presented, as the other three were academic and/or clinical. Characteristics of PHP cohort only available for *n* = 13 public and *n* = 5 professional.

† Evaluation survey submitted anonymously (n/d = not disclosed); ✓ = participated in listed activity; *x* = involved in prioritising follow-on research only.

Topics raised but not included in the final search strategy were: *identity* and *burden* (mentioned multiple times in discussion, but preliminary searches demonstrated that the terms retrieved unrelated content and were already covered by ‘*psychological dependency’*); *money* and *loss* (terms retrieved articles unrelated to self-management and loss was a major, implicit factor for most outcomes); and *massage and patient advocacy* (both retrieved articles unrelated to self-management and/or occupational therapy).

#### Interpreting the review findings

**Figure 3.**
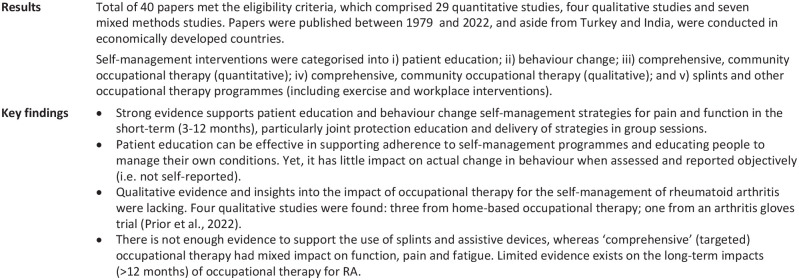
Results of the mixed methods systematic review (Gavin et al., 2022).

The review findings (Figure 3) were subsequently interpreted with the support from PHP during four online workshops with public partners (three workshops; *n* = 12) and occupational therapists (one workshop; *n* = 4 (Table 1)) respectively, who were presented results by the principal investigator (JG) for feedback. Public partners suggested presenting article publication dates when reporting the review findings to illustrate how the research has evolved over time. They also recognised a lack of reporting health inequalities and dietary advice in self-management programmes. For the latter, professional partners highlighted that biologics are the primary medical method for managing RA, whereas dietary guidance is only one of many self-management methods, and usually a lower priority than physical activity, medication and/or education.

The PHP interpretation workshops helped inform the dissemination strategy, publication writing, and follow-on research, which will be to develop a RA self-management programme. Public partners suggested that future self-management programmes should be personalised and targeted to the individual’s current needs (i.e. early or established RA). For example, one partner reported that the most impactful occupational therapy intervention they had received was a patient–practitioner consultation assessing which tasks they could and could not do with their hands at that time. Public partners welcomed telehealth interventions, particularly if the *patient–practitioner relationship* already existed, which concurred with our previous observations (Leese et al., 2022). Establishing rapport with healthcare professionals, involving carers/partners and supporting RA cohorts with other long-term conditions were identified as important considerations when developing follow-on self-management programmes.

Further salient topics were *personal relationships and the community environment*. For future occupational therapy interventions supporting self-management, cohabitation (e.g. with a partner, carer or housemate) should be considered when designing home or community-based interventions. Most suggested that living with others made maintaining self-care difficult sometimes, particularly during the COVID-19 pandemic, as their home environment was not always their personal space. However, the prospect of loneliness was much worse. Involving partners/carers in future interventions was valued by individuals, particularly given that partner involvement fosters understanding and awareness of the RA patient’s needs (Hewlett et al., 2019; Pow et al., 2018). Being responsive to the unforeseen needs of public partners is also important for researchers; herein, public needs related to altering meeting times, and given that PPI was online (via videocall or email), this afforded flexibility and reduced travel demands for our PHP partners.

*Feasibility* was another central topic. Professional partners considered that providing six to eight individual occupational therapy sessions (e.g. as in Macedo et al. (2009) would be unfeasible in the current post-COVID-19 climate. Initial telephone consultations, conducted after the COVID-19 outbreak, limited the patient–practitioner relationship (by hampering rapport building) but still allowed patient-centred care. Telehealth was described by public and professional partners as effective for activities of daily living problems but severely limited when conducting assessments, particularly of hand function and disability. Regarding outcomes, professional partners agreed that their overall focus is to improve function, particularly patients’ perception of and self-efficacy for improvement. Satisfaction was also important but rarely included and assessed in clinical research trials. All professional partners recommended Hewlett and colleagues (2019) programme and had adapted their fatigue management practices accordingly.

### Evaluation of public, professional and ECR involvement

#### Survey

Eleven surveys were completed, six by people living with RA (46% response rate), two by professionals (50%) and three by the ECRs involved in conducting the systematic review (100%) (JG, LR and JL). Two online evaluation workshops (*n* = 7) were subsequently convened, to (i) elaborate on the public survey responses (*n* = 5) and (ii) explore health professional partners’ experiences of involvement in the review (*n* = 2). Summary findings from the project evaluation were finally shared with the public and professional partners at project completion (see Table 1).

Our survey found that the public (*n* = 6) and occupational therapists (*n* = 2) *felt valued and heard* (topic 1), although one partner questioned patients’ impact on the systematic review’s outcomes. Overall, public partners considered that the survey was too late post-project (1 month) to allow accurate reflection, particularly in recollecting what they learnt from involvement. Learning related to a greater understanding of the evidence base (which some partners suggested is inaccessible for those outside research institutions), developing critical appraisal skills and discussing the applicability of evidence in real-life clinical practice. Partners agreed that PHPI should be involved throughout the research process, particularly in co-authoring publications and funding proposals. One public partner felt their novel contribution to the review was ‘*a patient’s view, my experience, my disability, and being in a minority*.’ Lived experience was the main contribution from public partners, and from professional partners, perspectives as an occupational therapist supporting patients to educate on self-management strategies.

**Figure 4.**
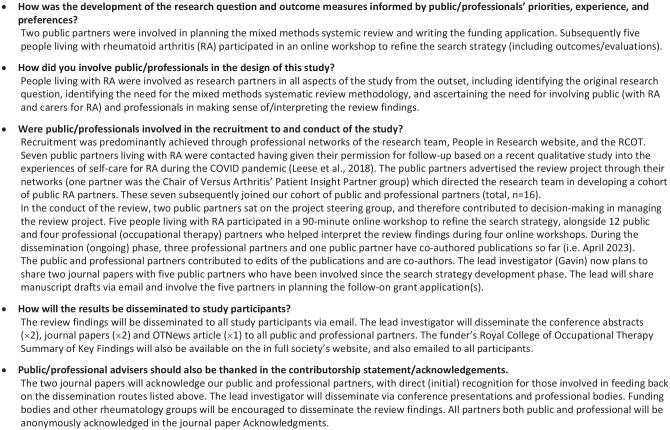
Public and health professional involvement in this mixed methods systematic review in GRIPP2 according to BMJ guidance.

In general, ECR co-authors considered that the challenges to their involvement in the review were: (i) unfamiliarity in reviewing mixed methods evidence, (ii) dedicating time (amongst managing other workload/responsibilities) and (iii) communicating with PHPI partners in a timely manner. They were enthusiastic about involving people living with, and partners/family and healthcare professionals caring for those with RA as PHPI contributors, as two had previously done this in projects, but not in a systematic review. Although PHPI was considered effectively integrated (Figure 4), the time needed for communicating and conducting involvement activities was underestimated. Lessons learnt related to the importance of planning PHPI pre-project, involving a project steering group, and integrating involvement throughout a systematic review project (specifically the value that public/professionals can add to literature reviewing and the specific tasks they can support).

#### Workshops (public and professional only)

Public partners (*n* = 5) considered that the researchers had involved them throughout the project and particularly valued the sharing of the review findings to enhance interpretation. Two partners suggested that this could be continued into the review’s dissemination by involving public/professional partners to review the plain English summary and promote it through their involvement in charities and community groups. One commented, ‘*I think the challenge for the public is not finding out about research studies. Better understanding is needed, and it needs to be clearer as to what is expected of the public, patient, or carer in plain English*’ (female with RA, 62 years, white British).

Professional partners (*n* = 2) valued learning from other occupational therapists nationwide, and beyond their daily practice (topic 2 – *sharing and learning*). They also benefitted from viewing the preliminary review findings in the interpretation phase, which gave them insights into the evidence base to inform future practice. All public and professional partners considered they were informed, with some specifying that there was sufficient time before meetings to prepare/review for activities: ‘. . .*clear and regular communication . . . knowing where the project is going without giving unnecessary information*’ (male, 33 years, British Pakistani). One experienced RA advocate commented that this is sometimes absent from projects: ‘. . .*involvement from the outset. . .clear focus of what you* [the researcher] *want from PPI and the objectives*’ (female, 52 years, British Indian). Role descriptions and clear expectations were deemed important, albeit one individual empathised that for research scientists, it is not always apparent where or how to include PHPI (topic 3 – *communication and listening for partnership building*).

Relating to topic 3, not knowing what is planned was deemed disruptive to PHPI and establishing trust in forging public partnerships, which was highly valued for follow-on research, ‘*. . .it’s about building relationships . . . it’s less transactional . . . people will be willing to work with you again*’ (female, 57 years, white Scottish). For this 12-month review project, we did not have sufficient time to forge trusting relationships with our PHP cohort. However, we did have an established relationship with our RA co-investigator (12–18 months previous), who we had collaborated with on another project (Leese et al., 2022). We recommend that researchers liaise with public and professional partners to involve them ‘longitudinally’, when conducting future-related projects. Online involvement was considered positive overall, but many individuals recommended having at least one in-person meeting (per year) during a project. One warned, ‘*we are missing the seldom seen voices’* (female, 52 years, British Indian) by holding only online meetings and questioned, ‘*how would you get that word over to different and wider communities?’*. Accessing charities and support groups was recommended for researchers in planning their studies. Concordant with the survey results, partners considered that the evaluation should have been circulated immediately post-project, from when their involvement ceased.

## Discussion

Our results present a novel perspective of the advantages and challenges of involving the public and professionals (herein rheumatology occupational therapists) in research, specifically in conducting a mixed methods systematic review. The research team believed that by involving PHP to inform the review search and in helping interpret findings, they could offer in-depth insights into the review’s main qualitative outcome, that is, the ‘lived experience’ of RA. Our evaluation of involving public and healthcare professionals in this systematic review demonstrated that PHPI is worthwhile and can be integrated and assessed throughout a review project.

Partners were involved in steering the project and evaluation, with one partner co-authoring a journal article and two professional partners co-authoring a charity magazine article. Based on the evaluation, the investigators acknowledged that greater involvement (beyond a single public partner co-investigator) earlier on was needed to bring broader perspectives. This would have supported further defining the review question and initially conceptualising the dissemination/impact strategy with UK partners using a ‘real-time’ co-production approach. For example, generating the research question in project planning, as a group of academics, clinicians and members of the public with lived experience offered different insights and experiences. Our RA advocate and co-investigator (ED) was highly experienced in research; however, as a Canadian resident, lacked familiarity with rheumatology services, charities and communities that UK partners could have shared.

Our public and professional partners reflected on what they learnt from participating in the review, and that it was a valuable experience in understanding the literature. Researchers were reminded of the need to invest time preparing involvement activities and communicating clearly in advance, particularly in seeking evaluation and feedback from public and professionals. Our public partners helped strengthen our search strategy and interpretation of the preliminary results from 40 articles selected for review. In refining the search strategy, interestingly, the terms suggested encompassed physical (e.g. body image) and mental concepts (e.g. self-esteem), but primarily social concepts (dependency, identity, stigma and isolation). Although measures to assess these social ‘outcomes’ are becoming more common and validated in healthcare research, most clinical research trials remain limited to assessing outcomes quantitatively (e.g. using numerical representations of pain, function and fatigue). Involving patients and professionals in interpretation afforded the reviewers insights into the nuances of living with RA and the type of self-management programmes they would like. Three topics were highlighted to consider when developing future RA self-management programmes: (i) the patient–practitioner relationship, (ii) personal relationships and community environments and (iii) feasibility (for RA patients and healthcare professionals). These align with the social concepts raised in the search strategy.

Public and professional partners thought that involvement should have been evaluated immediately after the reviewing and interpretation phases and not later (>4 weeks) in dissemination. We recommend that researchers apply this across research types, not only systematic reviews, to facilitate accurate and truthful feedback from partners. Previously Vale et al. (2012) found clear information and regular communication were important, in involving five cancer patients in their systematic review. Feedback was sought 1- and 2-years post-review, yet their formal evaluation came 5 years after the initial steering group meeting. Communication was valued in our review over 12 months, albeit for longer research programmes (>2–5 years), regular information provision is of greater importance to maintain partners’ interest and motivation.

Public involvement in occupational therapy research is becoming more common. However, few formal evaluations for involvement exist; those that do are anecdotal and predominantly focused on qualitative research involving public/patients and not professionals (Røssvoll et al., 2022). Our evaluation of PHPI contributes to the evidence base by reporting findings from a mixed methods systematic review involving people with RA and occupational therapists at critical stages of the review process. Our evaluation survey was completed by only 46% and 50% of our public and professional partners, respectively. However, all three ECR co-authors who liaised with PHP in conducting the review completed an evaluation survey. Furthermore, follow-on evaluation workshops afforded in-depth discussion of the anonymous survey responses and allowed us to generate themes for greater insights into the experiences of our public and professional partners. These workshops included a range of genders, ethnicities and ages representative of adults in England with RA and also involved specialist occupational therapists supporting people with RA.

Partners in our evaluation recommended co-authorship in publication and funding applications. This is widely accepted by UK national funding bodies such as the NIHR and Versus Arthritis, and in our discussion, publishing via Open Access (OA) journals was recognised as a priority (including securing funding for OA) to eliminate financial barriers for public and professional readers and reach broader audiences. A recommendation for maximising reach was to involve the public/professionals earlier in a project to map dissemination pathways (e.g. charities, community groups) and subsequently writing/reviewing the plain English summary (a mechanism for engaging different public). Our evaluation highlighted the importance of involving public partners in recruiting from local networks and community groups, particularly in ‘under-served’ populations. Well-crafted lay summaries and accessible media can help engage these excluded populations, but researchers must first work to establish trusted partnerships in public communities. In dissemination, researchers should include various activities, including writing groups or public workshops, to further involve different public groups based on their skills and knowledge (Giebel et al., 2019). Ultimately, PHPI in this review has influenced our practice, impacting our decisions on planning, delivering and communicating our research. Early follow-on work has included using PHP partnerships developed herein, to mobilise a regional PPI network to co-design methodology to explore local health inequalities for musculoskeletal care.

## Limitations

While PHPI was not included in the review selection, synthesis and analysis, we kept our network of partners aware of our progress and provided summaries of our review actions. Due to the project limitations (12-month funded review), we could not invest time and funding into upskilling partners but explained each step guided by our PRISMA flowchart. Regardless of the research type, investigators should engage the public and professionals in planning PHPI before a project commences. For example, in identifying activities for different types of involvement (e.g. advising, reviewing or data collection, co-interpreting the results), mapping dissemination pathways and impact planning (including highlighting relevant stakeholders and future opportunities). Finally, we acknowledge that we did not conduct a formal thematic analysis, nor quantitative survey evaluation. This article reports from PHPI, not a prospectively planned qualitative study, which would have included formal analyses. However, this article offers guidance on considerations for clinical academics in delivering meaningful public and professional involvement in research.

## Conclusion

This evaluation has demonstrated that involving the public and healthcare professionals in a mixed methods systematic review of RA self-management is feasible, worthwhile and provides benefits throughout the review process. Our workshops were valued, and deemed effective in promoting group learning and shared-practice, by both public and professional (occupational therapy) partners. This included evaluation workshops, which also highlighted the need for researchers to establish: roles and expectations with PHP partners; trust for partnership building; and PHPI in developing and implementing the dissemination strategy. Our ECRs learnt to prioritise time in planning and communicating public involvement activities during the review. Evaluations of involvement are important and should be factored into project timelines in the research planning phase, as timely evaluations offer more accurate and valuable PHP feedback. Finally, communication and regular involvement foster partnership building and support follow-on research.

**Key findings**

- PHPI in a mixed methods review is feasible and can support follow-on research.
- Timely evaluations are important for improving PHPI feedback.
- Co-authorship is mutually valued by PHP partners and researchers.

**What the study has added**

This evaluation adds to the evidence base exemplifying the value of involving the public and healthcare professionals in research, particularly within a mixed methods systematic review on the impact of occupational therapy in the self-management of rheumatoid arthritis.

## Supplemental Material

sj-docx-1-bjo-10.1177_03080226231219106 – Supplemental material for Public and professional involvement in a systematic review investigating the impact of occupational therapy on the self-management of rheumatoid arthritis Supplemental material, sj-docx-1-bjo-10.1177_03080226231219106 for Public and professional involvement in a systematic review investigating the impact of occupational therapy on the self-management of rheumatoid arthritis by James P Gavin, Laura Rossiter, Vicky Fenerty, Jenny Leese, Jo Adams, Alison Hammond, Eileen Davidson and Catherine L Backman in British Journal of Occupational Therapy

sj-docx-2-bjo-10.1177_03080226231219106 – Supplemental material for Public and professional involvement in a systematic review investigating the impact of occupational therapy on the self-management of rheumatoid arthritis Supplemental material, sj-docx-2-bjo-10.1177_03080226231219106 for Public and professional involvement in a systematic review investigating the impact of occupational therapy on the self-management of rheumatoid arthritis by James P Gavin, Laura Rossiter, Vicky Fenerty, Jenny Leese, Jo Adams, Alison Hammond, Eileen Davidson and Catherine L Backman in British Journal of Occupational Therapy
