# Supplementary material for: Public and professional involvement in a systematic review investigating the impact of occupational therapy on the self-management of rheumatoid arthritis
Source: Br J Occup Ther. 2023 Dec 30;87(4):201–12. doi: 10.1177/03080226231219106 (PMC12033777; doi:10.1177/03080226231219106)
Supplement: sj-docx-1-bjo-10.1177_03080226231219106 – Supplemental material for Public and professional involvement in a systematic review investigating the impact of occupational therapy on the self-management of rheumatoid arthritis [file sj-docx-1-bjo-10.1177_03080226231219106.docx]

**Evaluation survey questions – early career researchers (ECR)**

1. What was your initial understanding of public and patient involvement (PPI) prior to this review project?

2. How did you think PPI would be implemented in this systematic review prior to the project?

3a. Were there any challenges to being involved in this systematic review project as a ECR?

3b. Please explain your response

4. Did you feel there were any barriers to prevent you, or the research team, from responding or liaising with public, patient and professional partners in this project?

5. As an ECR, what did you learn in PPI from this project?

6. Was the final review of high quality? And do you feel the aims of the review were met, with regard to PPI?

7. Did you feel you were supported by the research team in working with public and professionals in this project?

8. Based on the review findings, what would you think the follow-up project should look at?

9. What do you think will be the impact of PPI in this project?

10. What changes could the research team make for future PPI in research?

11. As an ECR, what do you feel you have learnt in PPI during this review project?

12. Overall, were you satisfied with your involvement as an ECR?

**Supplementary Figure 1.** Public and health professional involvement in a mixed methods systematic review - evaluation survey (early career researcher [ECR] experiences).
