# Supplementary material for: Public and professional involvement in a systematic review investigating the impact of occupational therapy on the self-management of rheumatoid arthritis
Source: Br J Occup Ther. 2023 Dec 30;87(4):201–12. doi: 10.1177/03080226231219106 (PMC12033777; doi:10.1177/03080226231219106)
Supplement: sj-docx-2-bjo-10.1177_03080226231219106 – Supplemental material for Public and professional involvement in a systematic review investigating the impact of occupational therapy on the self-management of rheumatoid arthritis [file sj-docx-2-bjo-10.1177_03080226231219106.docx]

**Project steering group**

n=2 public (with RA)

n=1 occupational therapist

n=1 rheumatologist

n=1 academic (reviewer)

**August 2021**

**Evaluation (workshop)**

(n=5 public)

**Refining the search strategy** (n=5 public, with RA)

Cohort of public partners (n=13)

**Interpreting the review findings** (n=12 public*)

Cohort of professional partners (n=3)

**Interpreting the review findings** (n=4 professional)

Additional professional partner (n=1^†^)

**Evaluation (workshop)**

(n=2 professional)

Lack of time/availability (n=2)

**September 2021**

**June – August 2022**

**July – September 2022**

**Evaluation (anonymous survey)**

n=6 public

n=3 professional

n=3 ECR

**October – November 2022**

**Supplementary Figure 2.** Flow chart of public and professional participation at different stages of the mixed methods systematic review process.

Interpreting the review findings: *Public partners (n=12) were involved in three online workshops, each with n=4 people (public) living with, or caring for rheumatoid arthritis (RA). Professional partners (n=4) were involved in one interpretation workshop. ^†^One rheumatology occupational therapist joined the professional network for the interpretation only. ECR, early career researcher.
